# Beyond direct neighbourhood effects: higher-order interactions improve modelling and predicting tree survival and growth

**DOI:** 10.1093/nsr/nwaa244

**Published:** 2020-09-23

**Authors:** Yuanzhi Li, Margaret M Mayfield, Bin Wang, Junli Xiao, Kamil Kral, David Janik, Jan Holik, Chengjin Chu

**Affiliations:** Department of Ecology, State Key Laboratory of Biocontrol, School of Life Sciences, Sun Yat-sen University, Guangzhou 510275, China; School of Biological Sciences, The University of Queensland, Brisbane, Queensland 4072, Australia; Guangxi Key Laboratory of Plant Conservation and Restoration Ecology in Karst Terrain, Guangxi Institute of Botany, Guangxi Zhuang Autonomous Region and Chinese Academy of Sciences, Guilin 541006, China; Department of Ecology, State Key Laboratory of Biocontrol, School of Life Sciences, Sun Yat-sen University, Guangzhou 510275, China; Department of Forest Ecology, Silva Tarouca Research Institute, Brno 61200, Czech Republic; Department of Forest Ecology, Silva Tarouca Research Institute, Brno 61200, Czech Republic; Department of Forest Ecology, Silva Tarouca Research Institute, Brno 61200, Czech Republic; Department of Silviculture, Faculty of Forestry and Wood Technology, Mendel University in Brno, Brno 61300, Czech Republic; Department of Ecology, State Key Laboratory of Biocontrol, School of Life Sciences, Sun Yat-sen University, Guangzhou 510275, China

**Keywords:** pairwise interactions, indirect interactions, density-dependent, size- and distance-dependent, Zofin Forest Dynamic Plot

## Abstract

It is known that biotic interactions are the key to species coexistence and maintenance of species diversity. Traditional studies focus overwhelmingly on pairwise interactions between organisms, ignoring complex higher-order interactions (HOIs). In this study, we present a novel method of calculating individual-level HOIs for trees, and use this method to test the importance of size- and distance-dependent individual-level HOIs to tree performance in a 25-ha temperate forest dynamic plot. We found that full HOI-inclusive models improved our ability to model and predict the survival and growth of trees, providing empirical evidence that HOIs strongly influence tree performance in this temperate forest. Specifically, assessed HOIs mitigate the competitive direct effects of neighbours on survival and growth of focal trees. Our study lays a foundation for future investigations of the prevalence and relative importance of HOIs in global forests and their impact on species diversity.

## INTRODUCTION

The tremendous diversity of species on Earth has motivated a century of research into the mechanisms maintaining it. The question ‘*What determines patterns of species diversity?*’ is still among the big unknowns identified in the 125th anniversary issue of *Science* [1]. The interactions between the environment and living organisms and between the organisms themselves are known to play key roles in encouraging or discouraging species diversity [2]. Traditional studies of the mechanisms maintaining local patterns of diversity have focused overwhelmingly on direct interactions between organisms, with the ‘pairwise interaction’ considered as a fundamental unit in ecology [3–8]. Ecologists often take the reductionist approach that attempts to build up a complete understanding of complex natural systems by adding up all pairwise interactions [9,10]. However, complex natural systems composed of multiple interacting species often form interactive networks that cannot be easily reduced to pairwise interactions [11,12], foiling the reductionist approach. Thus, it is not surprising that models based on pairwise interactions alone often do a poor job of accurately explaining individual fitness [13,14], population dynamics [15,16] and the stability of complex systems [17,18]. This gulf between theory and reality has led to increased interest in higher-order interactions (HOIs).

There are two distinct definitions of HOIs in community ecology. Classical HOIs (or ‘hard’ HOIs), also termed ‘interaction modifications’ [19–22], are defined as the change of per capita effect of species *j* (the ‘transmitter’ of HOIs) on species *i* (the ‘receiver’ of HOIs) in the presence of species *k* (the ‘initiator’ of HOIs). By this definition, the HOIs of species *k* on species *i* through species *j* emerge when the transmitter (species *j*) has a plastic morphological or behavioural response to the initiator (species *k*) and this functional change of species *j* modifies its per capita effect on the receiver (species *i*) [23,24]. Importantly, the initiator (species *k*) or the transmitter (species *j*) can be the same species as the receiver (species *i*), and as such by this definition, HOIs can emerge in systems of two or more species [21,25]. Recently, HOIs have been further generalised as the non-additive effects of density (‘soft’ HOIs), including interspecific interaction modifications and intraspecific nonlinear density dependency [13,16,17,21,26]. The soft HOIs can be thought of as the modification of individual interactions such that the effect of individual*p* (the transmitter) on individual*m* (the receiver) is modified by individual*q* (the initiator), where individuals *m*, *p* and *q* can be the same or different species (i.e. HOI of *q* on *m* through *p*). From this perspective, it becomes clear that the seemingly different definitions of hard and soft HOIs can actually be reconciled at the individual level, and these individual-level HOIs may emerge in systems of three or more individuals of any number of species. Four-way or even higher-levels of HOIs, in which the interaction between two individuals is modified by two or more other individuals, are rarely considered in empirical studies (including ours) because of the huge number of parameters associated with them and the likelihood for diminishing returns on their inclusion. This is indeed the reason why these HOIs have only been explored theoretically [17,27].

The literature on HOIs from both theoretical and empirical perspectives is mostly based on Lotka-Volterra models investigating the role of HOIs in population dynamics [16–18,25,26,28]. Population dynamics are ultimately the result of individual survival, growth and reproduction. Therefore, the importance of HOIs in population dynamics is essentially the effects of HOIs on individual fitness (survival, growth and reproduction), and thus should also be considered in individual fitness models. Mayfield and Stouffer [13] developed a simple mathematical framework for incorporating HOIs into individual fitness models, and found that individual seed production could be better explained when HOIs were considered in herbaceous communities. Researchers studying forests have proposed many different indices for estimating the outcome of interactions between a focal tree and its neighbours (but only direct interactions), and tested such neighbourhood effects on survival and growth of focal trees [29–35]. More recent studies have focused on the role of functional traits and phylogeny in survival and growth of trees [36–39], but no study, to our knowledge, has tested the impact of individual-level HOIs on demographic rates of forest trees.

In the study of herbaceous plant communities [13], the direct and higher-order effects of neighbours on seed production of a focal individual were calculated as a function of the densities of these neighbours, which ignored individual variation within species (assuming neighbours have the same strength of direct and higher-order effects on the seed production of the focal individual). In forests, however, the direct effect of a neighbour on a focal tree is often assumed to be directly proportional to the size of the neighbour (usually quantified as diameter at breast height) and inversely proportional to the distance between the neighbour and the focal tree [29,33–35]. Given that HOIs modify direct interactions, we also expected HOIs to be size- and distance-dependent. In this study, we present a novel method of calculating size- and density-dependent individual-level HOIs for trees (Box 1) and test the importance of individual-level HOIs to tree survival and growth in a 25-ha Zofin Forest Dynamics Plot (ZFDP) in South Bohemia.

**Box 1. Size- and distance-dependent individual-level higher-order interactions (HOIs).**

**Figure 1.**
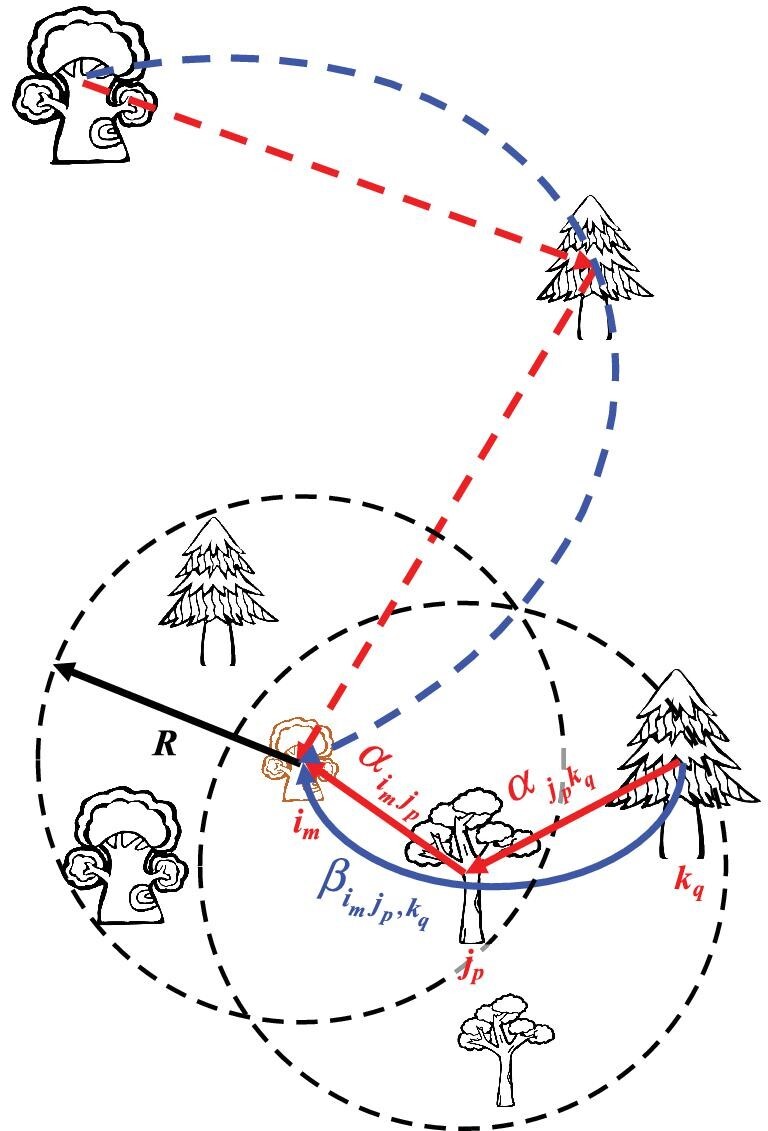
Direct (red arrows) and higher-order interactions (blue arrows) of neighbouring trees on a focal tree (the brown one). The parameter ${{\rm{\alpha }}_{{{{i}}_{{m}}}{{{j}}_{{p}}}}}$ quantifies the direct effect of a neighbour (individual *p* of species *j*) on the focal tree (individual *m* of species *i*). The direct interaction occurs only when a neighbour (*j_p_*) is located within a maximum radius (*R*) of *i_m_* (solid red arrows). The parameter ${{{\beta }}_{{{{i}}_{{m}}}{{{j}}_{{p}}},{{{k}}_{{q}}}}}$ quantifies the higher-order effect of a neighbour (individual *q* of species *k*) on the focal tree through another neighbour (individual *p* of species *j*). Higher-order interaction occurs only when *j_p_* is located within the maximum radius (*R*) of *i_m_* and *k_q_* is located within the maximum radius (*R*) of *j_p_* (solid blue arrows). Dashed arrows indicate direct interactions and higher-order interactions that are not considered when a neighbour is located outside the maximum radius (*R*) of the focal tree or its neighbour.

Consider a general case of *N* individual trees of *S* species (${{N\,\, = }}\mathop \sum \nolimits_{{{j = 1}}}^{{S}} {{{N}}_{{j}}}{{\,\,}}$, *N_j_* is number of individuals from species *j*) found around a focal tree (Fig. 1). The direct effect of a neighbour (*j_p_*, individual *p* of species *j*) on the focal tree (*i_m_*, individual *m *of species *i*) is ${{\rm{\alpha }}_{{{{i}}_{{m}}}{{{j}}_{{p}}}}}$, then direct effects of the *N* neighbours on *i_m_* (${{\rm D}}{{{\rm I}}_{{{{i}}_{{m}}}}}{{|[N]}}$) are the sum of their direct effects (Fig. 1, red arrows):

(1) \begin{eqnarray*} {\rm DI}_{{i_m}}|[N] = \sum\limits_{j = 1}^S {\sum\limits_{p = 1}^{{N_j}} {{\alpha _{{i_m}{j_p}}}} }. \end{eqnarray*}

The direct effect of a neighbour (*j_p_*) on a focal tree (*i_m_*), ${{\rm{\alpha }}_{{{{i}}_{{m}}}{{{j}}_{{p}}}}}$, is often assumed to be directly proportional to the size of its neighbour ($\rm {DBH}_{\it j_p})$ and inversely proportional to the distance between the neighbour and the focal tree (*d*[*i_m_*, *j_p_*]) [33,35], and is assumed to occur only when *j_p_* is located within a maximum radius (*R*) of *i_m_* (Fig. 1, solid red arrows):

(2) \begin{eqnarray*} {\alpha _{{i_m}{j_p}}} = \left\{ {\begin{array}{@{}*{2}{l}@{}} {{\alpha _{ij}} \cdot \frac{{\rm DBH_{{\it j_p}}^{\it u}}}{{d{{[{i_m},{j_p}]}^v}}}} {i\!\!f\,d[{i_m},{j_p}] < R}\\ 0 {i\!\!f\,d[{i_m},{j_p}] \ge R} \end{array}} \right.. \end{eqnarray*}

The parameter α_*ij*_ is the species-specific effect of species *j* on species *i*, which can be competitive (α_*ij*_ < 0) or facilitative (α_*ij*_ > 0). The parameters *u* and *v* determine the shape of the effect of the DBH and distance to the focal tree, respectively. For computational tractability, we set the distance between a neighbour and the focal tree (*d*[*i_m_*, *j_p_*]) to ∞ if it is greater than *R* to exclude the direct effect of a neighbour that is located outside a predetermined maximum radius (*R*) of *i_m_* (Fig. 1, dashed red arrows). Then direct effects of the *N* neighbours on *i_m_* (${{\rm D}}{{{\rm I}}_{{{{i}}_{{m}}}}}{{|[N]}}$) account for size and distance as:

(3) \begin{eqnarray*} {\rm DI}{_{{i_m}}}|[N] = \sum\limits_{j = 1}^S {{\alpha _{ij}} \cdot \left( {\sum\limits_{p = 1}^{{N_j}} {\frac{{\rm DBH_{\it {j_p}}^{\it u}}}{{d{{[{i_m},{j_p}]}^v}}}} } \right)}\\ {\left( {i\!\!f\,d[{i_m},{j_p}] \ge R,d[{i_m},{j_p}] = \infty } \right)}.\\ &&{\left( {i\!\!f\,d[{i_m},{j_p}] \ge R,d[{i_m},{j_p}] = \infty} \right)} \end{eqnarray*}

The higher-order effect of a neighbour (*k_q_*) on the focal tree (*i_m_*) through another neighbour (*j_p_*) is ${{\rm{\beta }}_{{{{i}}_{{m}}}{{{j}}_{{p}}},{{\,\,}}{{{k}}_{{q}}}}}$ (functional changes of *j_p_* in the presence of *k_q_* alter its effect on *i_m_*), then the higher-order effects of the *N* neighbours on *i_m_* (${{\rm HO}}{{{\rm I}}_{{{{i}}_{{m}}}}}{{|[\it N]}}$) are the sum of all higher-order effects of a neighbour on the focal tree through another neighbour (blue arrows):

(4) \begin{eqnarray*} {\rm HOI{_{{\it i_m}}}|[\it N] = \sum\limits_{j = 1}^S {\sum\limits_{k = 1}^S {\sum\limits_{p = 1}^{{N_j}} {\sum\limits_{q = 1}^{{N_k}} {{\beta _{{i_m}{j_p},{k_q}}}}}}}.} \end{eqnarray*}

The case when *j_p_* and *k_q_* are the same individual (*j* = *k* and *p* = *q*) is excluded because the higher-order effect of *j_p_* on *i_m_* through itself (${{\rm{\beta }}_{{{{i}}_{{m}}}{{{j}}_{{p}}},{{{j}}_{{p}}}}}$) is biologically nonsensical. The higher-order effect of *k_q_* on *i_m_* through *j_p_* (${{\rm{\beta }}_{{{{i}}_{{m}}}{{{j}}_{{p}}},{}{{{k}}_{{q}}}}}$) depends on both the direct effect of *k_q_* on *j_p_* (${{\rm{\alpha }}_{{{{j}}_{{p}}}{{{k}}_{{q}}}}}$) and the direct effect of j_p_ on *i_m_* (${{\rm{\alpha }}_{{{{i}}_{{m}}}{{{j}}_{{p}}}}}$), and thus occurs only when *j_p_* is located within the maximum radius of *i_m_* and *k_q_* is located within the maximum radius of *j_p_* (Fig. 1, solid blue arrows):

(5) \begin{eqnarray*} {{\beta _{{i_m}{j_p},{k_q}}} = \left\{ {\begin{array}{@{}*{2}{l}@{}} {\beta _{ij,k}} \cdot \frac{{DBH_{{j_p}}^u}}{{d{{[{i_m},{j_p}]}^v}}} \cdot \frac{{DBH_{{k_q}}^u}}{{d{{[{j_p},{k_q}]}^v}}}&\\ \quad\quad{i\!\!f\,d[{i_m},{j_p}] < R\,\,and\,\,d[{j_p},{k_q}] < R}\\ 0 {i\!\!f\,d[{i_m},{j_p}] } {\ge R\,\,or\,\,d[{j_p},{k_q}] {\ge} R} \end{array}} \right.\!\!\!.} \end{eqnarray*}

The parameter β_*ij, k*_ is the species-specific higher-order effect of species *k* on species *i* through species *j*, which may intensify direct competition (α_*ij*_ < 0 and β_*ij, k*_ < 0), intensify direct facilitation (α_*ij*_ > 0 and β_*ij, k*_ > 0), weaken direct competition (α_*ij*_ < 0 and β_*ij, k*_ > 0) or weaken direct facilitation (α_*ij*_ > 0 and β_*ij, k*_ < 0). The distances *d*[*i_m_*, *j_p_*] and *d*[*j_p_*, *k_q_*] are also set to ∞ if they are greater than *R* to exclude the higher-order effect when a neighbour is located outside the maximum radius (*R*) of the focal tree or the intermediary tree (Fig. 1, dashed blue arrows). We note here that the higher-order effect of *k_q_* on *i_m_* through *j_p_* (${{\rm{\beta }}_{{{{i}}_{{m}}}{{{j}}_{{p}}},{{{k}}_{{q}}}}}$) is different from the higher-order effect of *j_p_* on *i_m_* through *k_q_* (${{\rm{\beta }}_{{{{i}}_{{m}}}{{{k}}_{{q}}}{{,\,\,}}{{{j}}_{{p}}}}}$) when size and distance are incorporated (*u*≠0 and *v*≠0). The higher-order effects of the *N* neighbours on *i_m_* (${\rm{HO}}{{\rm{I}}_{{{{i}}_{{m}}}}}{{|[N]}}$) account for size and distance as:

(6) \begin{eqnarray*} {\rm {HOI}{_{{i_m}}}|[N]} = {\sum\limits_{j = 1}^S {\sum\limits_{k = 1}^S {{\beta _{ij,k}}}}}\\ {{{\cdot \left( \sum\limits_{p = 1}^{{N_j}} \sum\limits_{q = 1}^{{N_k}} {\frac{{{\rm DBH}_{{j_p}}^u}}{{d{{[{i_m},{j_p}]}^v}}} \cdot \frac{{{\rm DBH}_{{k_q}}^u}}{{d{{[{j_p},{k_q}]}^v}}}} \right)} }}\\ \times\left({\begin{array}{@{}l@{}} i\!\!f\,d[{i_m},{j_p}] \ge R,d[{i_m},{j_p}] = \infty \\ i\!\!f\, d[{j_p},{k_q}] \ge R,d[{j_{mp}},{k_q}] = \infty \end{array}} \right). \end{eqnarray*}

## RESULTS

### Evidence of HOIs in individual survival and growth

The selected optimum *u* and *v* based on *R*-squared were the same as those based on likelihood for the survival and growth of Beech and Spruce in each maximum radius case (*R* = 10 m, 20 m and 30 m) (Table S1). The results for evaluations and comparisons of the three classes of models (SIZE, SIZE+DI and SIZE+DI+HOI) at the optimum *u* and *v* were generally the same in each maximum radius (Tables 1, S2 and S3), and thus we only report results at *R* = 20 m (Table 1) in the main text. Goodness-of-fit approaches (both R-squared and likelihood) showed a significant improvement in model fit for HOI-inclusive models over the SIZE and SIZE+DI models (Table 1). The Akaike's Information Criteria (AIC) comparisons of model parsimony indicated that HOI-inclusive models were the most parsimonious for survival and growth of Beech and Spruce despite penalties for large numbers of model terms (Table 1). Bayesian Information Criteria (BIC), which has a stronger penalty for each additional model term than AIC, still supported the HOI-inclusive models as the most parsimonious, except for growth of Spruce (Table 1). The k-fold cross validations further supported the HOI-inclusive models which had the lowest root mean squared error (RMSE) and mean absolute error (MAE) for survival and growth of both Beech and Spruce. Support for the HOI-inclusive models was robust when we repeated the analyses by categorising neighbours as large trees (DBH > 10 cm) and small trees (DBH ≤ 10 cm) instead of categorising them by species identity (Appendix S1 and Table S4).

**Table 1.** Evaluations of model performance based on the parsimony tests and repeated k-fold cross validations (10 folds and 10 repeats) in case of optimum *u* and *v* and Radius = 20 m.

Radius	Species	Response	Model	*u*	*v*	Para	Samples	R^2^	logLik	AIC	BIC	RMSE	MAE
20 m	Beech	Survival	SIZE	–	–	4	38 798	0.011	−1433	2874	2908	0.078	0.012
			SIZE + DI	0.2	0	6	38 798	0.028	−1409	2830	2881	0.078	0.012
			SIZE + DI + HOI	0.5	0.8	10	38 798	**0.062**	−**1360**	**2739**	**2825**	**0.078**	**0.012**
		Growth	SIZE	–	–	2	28 845	0.180	−35 775	71 557	71 582	0.836	0.671
			SIZE + DI	1.3	0.8	4	28 845	0.244	−34 611	69 231	69 272	0.803	0.641
			SIZE + DI + HOI	1	0.8	8	28 845	**0.252**	−**34 443**	**68 904**	**68 978**	**0.799**	**0.637**
	Spruce	Survival	SIZE	–	–	4	1058	0.099	−239	486	506	0.244	0.121
			SIZE + DI	1	0.5	6	1058	0.219	−207	427	457	0.234	0.111
			SIZE + DI + HOI	0.5	0.2	10	1058	**0.276**	−**192**	**405**	**454**	**0.233**	**0.108**
		Growth	SIZE	–	–	2	692	0.181	−967	1941	1954	0.978	0.788
			SIZE + DI	0.9	1	4	692	0.345	−890	1790	1813	0.878	0.692
			SIZE + DI + HOI	1	0.9	8	692	**0.361**	−**881**	**1781**	1821	**0.873**	**0.689**

Optimum *u* and *v* were selected for models with the highest R-squared and likelihood (Table S1). For the parsimony tests, AIC and BIC that were two or more points less than the next best model were considered as a meaningful improvement in in-sample performance. Models with lower RMSE and MAE computed from cross validations had better out-of-sample performance. The numbers in bold indicate that HOI-inclusive models had best performance based on AIC, BIC, RMSE or MAE. Results for the Radius = 10 m and 30 m are presented in Tables S2 and S3.

### Direct and higher-order effects on individual survival and growth

**Figure 2.**
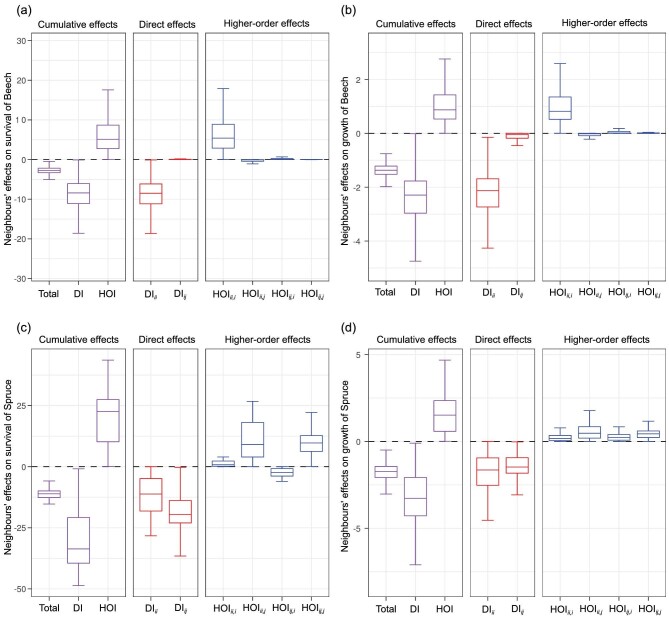
Cumulative (purple), direct (red) and higher-order (blue) effects on the survival (a and c) and growth (b and d) of each focal tree of *Fagus sylvatica* (Beech, a and b) and *Picea abies* (Spruce, c and d). Total indicates cumulative effects of all neighbours including both direct and higher-order effects. DI includes the direct effects of all neighbours including intraspecific direct effects (DI_*ii*_) and interspecific direct effects (DI_*ij*_). HOI includes the higher-order effects of all neighbours including intraspecific higher-order effects of conspecifics (HOI*_ii,__i_*), intraspecific higher-order effects of heterospecifics (HOI*_ij,__j_*) and interspecific higher-order effects (HOI*_ii,__j_* and HOI*_ij,__i_*). One boxplot represents the distribution of neighbourhood effects for all focal trees of a species. Boxplots above (or below) zero indicate that the effects are facilitative (or competitive) for all trees of a species, while boxplots crossing the zero line indicate that the effects are facilitative for some trees but competitive for others of a species.

The neighbourhood effects on the survival of Beech (Fig. 2a) were almost the same as the effects on growth of Beech (Fig. 2b). The survival probability and growth rate of Beech were lower for trees with neighbours within 20 m than trees growing in the absence of neighbours within 20 m as a result of total competitive neighbourhood effects. The total competitive neighbourhood effects resulted from stronger competitive net direct effects (DI) relative to facilitative net higher-order effects (HOI). The competitive direct effects were almost entirely from intraspecific direct effects (DI*_ii_*, DI of Beech on Beech) with weak interspecific direct effects (DI*_*ij*_*, DI of Spruce on Beech). The facilitative higher-order effects mainly stemmed from intraspecific HOIs of conspecifics (HOI*_ii,__i_*, HOI of one Beech on another Beech through the third Beech), while the other types of HOIs (HOI*_ii,__j_*, HOI*_ij,__i_* and HOI*_ij,__j_*) were negligible.

The cumulative neighbourhood effects on survival (Fig. 2c) and growth (Fig. 2d) of Spruce were basically the same (competitive total, competitive DI and facilitative HOI) as those on Beech. The intraspecific direct effects (DI*_ii_*, DI of Spruce on Spruce) and interspecific direct effects (DI*_*ij*_*, DI of Beech on Spruce) contributed almost equally to net direct effects. For survival of Spruce, HOIs resulted from facilitative HOI*_ii,__j_* (HOI of a Beech on a Spruce through another Spruce) and HOI*_ij,__j_* (HOI of a Beech on a Spruce through another Beech) and a relatively weaker competitive HOI*_ij,__i_* (HOI of a Spruce on another Spruce through a Beech). For growth of Spruce, HOIs were made up of the four almost equal

types of facilitative HOIs (HOI*_ii,__i_*, HOI*_ii,__j_*, HOI*_ij,__i_* and HOI*_ij,__j_*).

## DISCUSSION

The urgency and prospect of understanding the dynamics of complex natural systems has been a central motivation of recent work on HOIs [13,16–18,20,26,51]. Although theoretical research has highlighted the importance of HOIs in population dynamics, species coexistence and system stability by modelling per capita growth rates with or without the inclusion of HOIs [16–18], few studies have investigated the impact of HOIs on individual fitness outcomes (survival, growth and reproduction; but see [13]). In this study, we first developed a novel method for calculating size- and distance-dependent higher-order interactions based on neighbourhood analyses (Box 1), and then applied this method to the ZFDP census data to test the role of HOIs on survival and growth of trees. We found that HOI-inclusive models were always better at capturing neighbourhood effects on survival and growth of two temperate tree species (*Fagus sylvatica*—Beech and *Picea abies—*Spruce, Table 1), providing clear evidence of the occurrence and importance of HOIs in this forest system. This result was consistent with recent findings from herbaceous communities [13], and taken together they provide strong empirical evidence that HOIs had the same importance in individual fitness outcomes (survival, growth and reproduction) as found for population dynamics.

Following on these findings, we then explored how HOIs affected the survival and growth of trees. The net higher-order effects on survival and growth of Beech and Spruce were all facilitative (Fig. 2), but their strengths were weaker relative to the strengths of competitive direct effects (thus the total neighbourhood effects were negative, Fig. 2). Therefore, most previous studies of tree survival and growth (e.g. [29,30,32–34,36,38,45]) might underestimate the neighbourhood direct competitive effects without considering such facilitative higher-order effects. A study of annual plants has found facilitation to be systematically weaker than direct competition [52], but no previous studies have shown this difference to be mediated by HOIs. Such facilitative higher-order effects were also found in herbaceous communities affecting individual seed production [13] and population growth [26]. A facilitative higher-order effect (e.g. ${{\rm{\beta }}_{{{{i}}_{{m}}}{{{j}}_{{p}}},{{{k}}_{{q}}}}}$) emerges when individual *j_p_* has a plastic trait response in the presence of individual *k_q_* and this functional change of individual *j_p_* suppresses its competitive effect on focal individual *i_m_*. However, no study to date has tested which traits and how they mediate the HOIs in plant communities, which should be an important next step to understand the HOIs and their impact on species diversity.

The sources of direct and higher-order effects were different for Beech and Spruce, probably because of their spatial distributions and abundances (Fig. S1). Beech had over 50 000 trees distributed almost everywhere within the plot, whereas Spruce had only about 1000 trees distributed aggregately in a few places. Most Beech trees had no or few heterospecific neighbours (Spruce), thus the survival and growth of Beech were mostly reduced by intraspecific direct interactions (DI*_ii_*, direct effects of Beech on Beech). In contrast, most Spruce trees had both conspecific neighbours (Spruce) and heterospecific neighbours (Beech), thus the survival and growth of Spruce were reduced almost equally by intraspecific direct interactions (DI*_ii_*, direct effects of Spruce on Spruce) and interspecific direct interactions (DI*_*ij*_*, direct effects of Beech on Spruce). The competitive direct effects led to mostly facilitative higher-order effects (except for higher-order effect of a Spruce on another Spruce through a Beech, HOI*_ij,__i_* in Fig. 2c) in a case of ‘*the enemy of my enemy is my friend*’. More precisely, a neighbour of a focal tree is an ‘enemy’ or a ‘friend’ depending on its net effects of competitive direct effects and facilitative higher-order effects. The different types of HOIs (HOI*_ii,__i_*, HOI*_ii,__j_*, HOI*_ij,__i_* and HOI*_ij,__j_*) were not equally important for either species, especially for survival and growth of Beech in which there was barely any signal from any form of HOI other than HOI*_ii,__i_*. Findings for these tree species were quite different from the results from a diverse annual plant system in which no one type of HOI was more important than any other [13]. It would be interesting to explore this pattern across more systems to determine whether the dominance of specific types of HOIs decreases with increasing species diversity.

In our analyses, direct and higher-order interactions were size- (shape parameter *u*) and distance-(shape parameter *v*) dependent, and the optimum parameters *u* and *v* were selected for survival and growth of Beech and Spruce (Table S1), respectively. The optimum size shape parameter *u* for survival and growth of Beech and Spruce were ≤1, around half of previously reported *u* values, which were close to 2—the case of neighbourhood effects scaled to basal area of neighbours [33,45]. Neighbourhood direct and higher-order effects decayed rapidly with distance at optimum *v* and our defined radii were sufficient to capture the effects of neighbourhood interactions (Table S1 and Fig. S2), except for survival of Spruce at R = 10 m (*v* = 0.0) and 20 m (*v* = 0.2). This exception was in accordance with our expectations because an important source of Spruce mortality was the monophagous bark beetle (*Ips typographus*) and its infestation effects went far beyond 20 m. It might be more reasonable to set radius differently for each focal tree considering that larger trees may have longer interaction distance than smaller ones; however, this would make our models overly complicated so we used a fixed radius for all focal trees in our study. Therefore, size and distance did impact the strength of direct and higher-order interactions in forests, which were assumed to be size- and distance-independent (a special case of *u *= 0 and *v *= 0) for annual plants [13]. Further studies are needed to determine whether the importance of distance on HOIs found in our study is also evident in annual systems or if there are inherent differences in systems mediated by different limiting resources, statures or longevity.

By categorising neighbours as large trees (DBH > 10 cm) and small trees (DBH ≤ 10 cm), we further explored whether the direct effects and higher-order effects on survival and growth of Beech and Spruce come mainly from a few large trees or many small trees (Figs S3 and S4). The neighbourhood conspecific direct effects and higher-order effects on survival and growth of Beech (Fig. 2a and b), were split approximately equally between small and large trees (Fig. S5a and S5b). The same pattern was observed for growth of Spruce (Fig. S5d), but not for survival of Spruce in which neighbourhood direct effects and higher-order effects were largely caused by small trees (Fig. S5c). Although small trees have smaller per capita effects than large trees (optimum *u* > 0, directly proportional to their size), they can act in aggregate to have substantial direct and higher-order effects or even greater effects than large trees on survival and growth of their neighbours. These results highlighted the ecological importance of small trees in forest systems [53,54], which are often overlooked because of their small contributions to total forest biomass and timber extraction [55,56].

Despite low diversity of the forest system, our study still provides strong empirical evidence for the operation of individual-level HOIs in a natural forest system and lays the foundation for future investigations of the prevalence and relative importance of HOIs in more diverse forest communities. As the potential number of HOI terms using our current method increases with the square number of species which hinders its application in species-rich communities, additional studies are needed to identify systematic and ecologically grounded reasons for excluding certain types of model terms to allow this approach to be used effectively in diverse systems. For example, it may prove useful to categorise interacting individuals to a few ecologically meaningful groups by their sizes (large and small trees), life forms, functional traits or phylogenies rather than by their species identities, as this would greatly reduce the number of HOI terms in these models. Further, environmental factors and functional traits that are known to affect the individual fitness [57,58] should be integrated into future studies for more accurate assessments of HOIs and more insights into the mechanisms of HOIs.

## MATERIAL AND METHODS

### Study site

The Zofin Forest Dynamics Plot (ZFDP) is located in the Novohradské Hory Mts., South Bohemia (48°40′N14°42′E) within a strictly protected forest reserve. With a total area of 25 ha (500 m × 500 m), the ZFDP is positioned within a 75-ha research area that has been intensely studied since 1975. The mean annual temperature for the plot is 6.2°C and the mean annual precipitation total is 866 mm [40]. The ZFDP was established and incorporated into the Forest Global Earth Observatory (ForestGEO, https://www.forestgeo.si.edu) in 2012 (first census) and re-censused in 2017, following the ForestGEO forest census protocol [41]. In brief, all free-standing living woody stems with diameter at breast height (DBH) of at least 1 cm were tagged, mapped, measured and identified to species. In the 2012 census of ZFDP, 11 species were recorded. *Fagus sylvatica* (Beech) was the most dominant species, with 58 575 trees, and *Picea abies* (Spruce) was the second abundant species, with 1245 trees. All other species in this plot were represented by <100 trees and were not included in our analyses. Therefore, in this study, we tested how HOIs influence the survival (status in 2017 census) and growth (DBH increment between the 2012 and 2017 censuses) of the two dominant tree species (spatial distributions shown in Fig. S1).

### Calculating size- and distance-dependent direct and higher-order interactions

Using the ZFDP 2012 census data, we calculated size- and distance-dependent direct interactions and HOIs for each tree of the two focal species (Beech and Spruce) with the method presented in Box 1. In a simple case of two species, there were only two types of direct interactions (equation 3, direct effects of Beech and Spruce on a focal tree) and four types of HOIs (equation 6, HOI of a Beech on a focal tree through another Beech, HOI of a Beech on a focal tree through a Spruce, HOI of a Spruce on a focal tree through a Beech, and HOI of a Spruce on a focal tree through another Spruce). The simple diversity structure of this forest made it an ideal system to examine the importance of HOIs with a small number of parameters (six total), which provided robust proof-of-concept before applying this approach to a more diverse system (as the number of potential HOI terms in equation 6 will increase with the square of the number of species). Rather than setting *R*, *u* and *v* arbitrarily, as in recent studies [36,42,43], we explored the parameter space by calculating the direct interaction and HOI terms in 1323 cases with all combinations of three maximum radius values (*R* = 10 m, 20 m and 30 m), 21 size shape parameter values (*u* = 0, 0.1, 0.2, …, 2) and 21 distance shape parameter values (*v* = 0, 0.1, 0.2, …, 2). We did not consider trees at the edge of the plot (X < 2*R* or X > 500–2*R* or Y < 2*R* or Y > 500–2*R*) as focal trees because their neighbours or their neighbours’ neighbours (for HOIs) were often located outside the surveyed plot. These calculations relied on a matrix containing information about each focal tree and its neighbours within the maximum radius (*R*) using the ‘bigmemory’ package in R [44].

### Inclusion of HOIs into survival and growth models

In past studies, only direct effects of neighbours (equation 3) have been included in tree survival and growth models [33,34,36,38,45]. Here, we add HOIs (equation 6) to these models to explore whether doing so improves our ability to model and predict the survival and growth of trees. The survival probability of a focal tree in the presence of *N* neighbours (${\rm{Surviva}}{{\rm{l}}_{{{{i}}_{{m}}}}}{{|[N]}}$) is modelled as a function of its species identity (λ_*i*_), size (DBH) and neighbourhood direct effects [29,30], as well as the neighbourhood higher-order effects:

(7) \begin{eqnarray*} &&{\rm Survival}{_{{i_m}}}|\left[ N \right] &=&\\ &&\frac{1}{{1 + {e^{{\lambda _i} + {\gamma _1} \cdot DBH_{{i_m}}^{ - 1} + {\gamma _2} \cdot DB{H_{{i_m}}} + {\gamma _3} \cdot DBH_{{i_m}}^2 + D{I_{{i_m}}}|\left[ N \right] + {\rm HOI}{_{{i_m}}}|\left[ N \right]}}}}. \end{eqnarray*}

The size effect of the focal tree on its survival probability is modelled as γ_1_DBH^−1^ + γ_2_DBH + γ_3_DBH^2^, where the hyperbolic transformation of diameter (DBH^−1^) is hypothesised to track both the large mortality rates of small trees and the rapid decline in mortality rates associated with larger diameter trees, and the terms DBH and DBH^2^ are thus included to model the U-shaped senescence effect [30]. The growth of a focal tree (increment in DBH between the two census) in the presence of *N* neighbours (${\rm{Growt}}{{\rm{h}}_{{{{i}}_{{m}}}}}{{|[N]}}$) is modelled as a product of its potential growth (*G*_*pot,i*_, in the absence of neighbours), size and neighbourhood direct effects [36,38], as well as the neighbourhood higher-order effects:

(8) \begin{eqnarray*} \rm{Growth}{_{{i_m}}}|\left[ N \right] &=& {G_{pot,i}}\cdot \textrm{DBH}_{{i_m}}^\gamma\cdot {e^{D{I_{{i_m}}}|\left[ N \right]}}\\ \cdot\, {e^{HO{I_{{i_m}}}|\left[ N \right].}} \end{eqnarray*}

### Model fitting and evaluations

Individual survival (living status in 2017 census) is a discrete binary event, with value 0 (dead) or 1 (alive). Therefore, we conducted a logistic regression model to test the effects of tree size, direct and higher-order interactions on the survival probability of focal individuals for each species (equation 7). A log-transformation of equation 8 led to a linearised model of growth, so simple linear regressions were performed to model the effects of tree size, direct and higher-order interactions on the growth of individuals for each species. Whether or not to include the complex HOIs in the survival and growth models is a particular example of the ‘bias-variance trade-off’ [46,47]. Not only can complexity reduce uncertainty (variance) by tuning the model to match observations, but it also can increase the danger of ‘over-fitting’, leading to poor predictions under novel conditions (bias). To avoid the ‘over-fitting’ problem, we evaluated the performance of the HOI-inclusive models and two alternative classes of models as subsets of the HOI-inclusive models (SIZE+DI+HOI): tree size and direct interactions models (SIZE+DI) and tree size only models (SIZE). We first conducted regressions for the three classes of models in 441 combinations of 21 size shape parameter values (*u*) and 21 distance shape parameter values (*v*) and selected optimum *u* and *v* at which the SIZE + DI and SIZE + DI + HOI models were best fitted (highest R-squared or likelihood) in each maximum radius (*R* = 10 m, 20 m and 30 m). It should be noted that optimum *u* and *v* are not available for the SIZE models because they do not contain the parameters *u* and *v*. In case of optimum *u* and *v* in each maximum radius, we tested whether the HOI-inclusive model was most parsimonious using AIC and BIC comparisons, which statistically penalise for increased model complexity. We further conducted repeated k-fold cross validations (10 folds and 10 repeats) for the three classes of models using the ‘caret’ package [48] and tested whether the HOI-inclusive model had the best predictive performance as determined by the lowest RMSE and MAE. Finally, we used modified partial-residual plots to assess the relative magnitude and direction (competitive or facilitative) of neighbourhood cumulative effects, direct effects and higher-order effects [13,49]. Specifically, we multiplied each direct and higher-order interaction term by the corresponding coefficients estimated in the best-fit model, respectively. All the above mentioned calculations and statistical analyses were conducted in R 3.6.1 [50].

## DATA ACCESSIBILITY STATEMENT

Full census data for the Zofin forest plot are available upon reasonable request from the ForestGEO data portal: http://ctfs.si.edu/datarequest/.

## Supplementary Material

nwaa244_Supplemental_File
