# Supplementary material for: Beyond direct neighbourhood effects: higher-order interactions improve modelling and predicting tree survival and growth
Source: Natl Sci Rev. 2020 Sep 23;8(5):nwaa244. doi: 10.1093/nsr/nwaa244 (PMC8288344; doi:10.1093/nsr/nwaa244)
Supplement: nwaa244_Supplemental_File [file nwaa244_supplemental_file.pdf]

Supporting Information

Appendix S1 Categorising neighbours as large and small trees

In the main text, the neighbours of a focal tree were categorised by species identity (*Fagus sylvatica* and *Picea abies*) and assumed that different neighbours had different direct and higher-order effects on focal trees. We also categorised neighbours as large trees ($DBH >$ 10 cm) and small trees ($DBH \leq$ 10 cm) to see these direct and higher-order interactions are mainly from many small trees or from few large trees (Fig. S3). The spatial distribution of large (L) and small trees (S) within the 25-ha Zofin Forest Dynamic Plot (ZFDP) were displayed in Fig. S4. For N individuals ($N = N_L + N_S$, N_L is number of large trees and Ns is number of small trees) around a focal tree (i_m, individual m of species i), their direct effects on i_m ($\mathrm{DI}_{\mathrm{i_m}}$|[N]) can be classified into direct effects of large trees (DI_{iL}) and small trees (DI_{iS}) on the focal tree of species i:

$$DI_{i_m} \,|\, [N] = DI_{iL} + DI_{iS} = \alpha_{iL} \cdot \left(\sum_{p=1}^{N_L} \frac{DBH_{L_p}^{u}}{d[i_m, L_p]^{v}} \right) + \alpha_{iS} \cdot \left(\sum_{p=1}^{N_S} \frac{DBH_{S_p}^{u}}{d[i_m, S_p]^{v}} \right).$$

The higher-order effects of the N neighbours on i_m ($\mathrm{HOI}_{\mathrm{i_m}}$|[N]) can be classified into higher-order effects of large trees on focal tree in the presence of other large trees ($HOI_{iL,L}$), higher-order effects of large trees on focal tree in the presence of small trees ($HOI_{iL,S}$), higher-order effects of small trees on focal tree in the presence of large trees ($HOI_{iS,L}$), higher-order effects of small trees on focal tree in the presence other small trees ($HOI_{iS,S}$):

$$\begin{aligned} HOI_{i_m} \,|\, [N] &= HOI_{iL,L} + HOI_{iL,S} + HOI_{iS,L} + HOI_{iS,S} \\ &= \beta_{iL,L} \cdot \left(\sum_{p=1}^{N_L} \sum_{q=1}^{N_L} \frac{DBH_{L_p}^{u}}{d[i_m, L_p]^{v}} \cdot \frac{DBH_{L_q}^{u}}{d[L_p, L_q]^{v}} \right) + \beta_{iL,S} \cdot \left(\sum_{p=1}^{N_L} \sum_{q=1}^{N_S} \frac{DBH_{L_p}^{u}}{d[i_m, L_p]^{v}} \cdot \frac{DBH_{S_q}^{u}}{d[L_p, S_q]^{v}} \right) \\ &+ \beta_{iS,L} \cdot \left(\sum_{p=1}^{N_S} \sum_{q=1}^{N_L} \frac{DBH_{S_p}^{u}}{d[i_m, S_p]^{v}} \cdot \frac{DBH_{L_q}^{u}}{d[i_p, L_q]^{v}} \right) + \beta_{iS,S} \cdot \left(\sum_{p=1}^{N_S} \sum_{q=1}^{N_S} \frac{DBH_{S_p}^{u}}{d[i_m, S_p]^{v}} \cdot \frac{DBH_{S_q}^{u}}{d[S_p, S_q]^{v}} \right). \end{aligned}$$

The following analyses were conducted following the methods in the main text and the results were displayed in Table S2 and Fig. S5.

Table S1 The optimum tree size (*u*) and distance (*v*) parameters at which the size and direct interactions models (SIZE+DI) and the HOIs-inclusive models (SIZE+DI+HOI) in 441 combinations of 21 size shape parameter values ($u = 0, 0.1, 0.2, \ldots, 2$) and 21 distance shape parameter values ($v = 0, 0.1, 0.2, \ldots, 2$) had highest R-squared and likelihood for the survival and growth of Beech (*Fagus sylvatica*) and Spruce (*Picea abies*) in each maximum radius case ($R =$ 10 m, 20 m and 30 m). McFadden pseudo-R-squared was given for logistic regressions of survival models.

Radius	Species	Response	Models	R-squared_u	R-squared_v	R-squared	logLik_u	logLik_v	logLik
10 m	FS	survival	SIZE+DI	1.2	0.3	0.009	1.2	0.3	-2246
			SIZE+DI+HOI	0.6	0.3	0.041	0.6	0.3	-2173
		growth	SIZE+DI	1.1	0.6	0.230	1.1	0.6	-42331
			SIZE+DI+HOI	0.9	0.7	0.238	0.9	0.7	-42148
	PA	survival	SIZE+DI	1.1	0.3	0.189	1.1	0.3	-227
			SIZE+DI+HOI	0.8	0.0	0.222	0.8	0.0	-218
		growth	SIZE+DI	0.8	0.8	0.342	0.8	0.8	-967
			SIZE+DI+HOI	0.8	0.7	0.355	0.8	0.7	-959
20 m	FS	survival	SIZE+DI	0.2	0.0	0.028	0.2	0.0	-1409
			SIZE+DI+HOI	0.5	0.8	0.062	0.5	0.8	-1360
		growth	SIZE+DI	1.3	0.8	0.244	1.3	0.8	-34611
			SIZE+DI+HOI	1.0	0.8	0.252	1.0	0.8	-34443
	PA	survival	SIZE+DI	1.0	0.5	0.219	1.0	0.5	-207
			SIZE+DI+HOI	0.5	0.2	0.276	0.5	0.2	-192
		growth	SIZE+DI	0.9	1.0	0.345	0.9	1.0	-890
			SIZE+DI+HOI	1.0	0.9	0.361	1.0	0.9	-881
30 m	FS	survival	SIZE+DI	0.2	0.0	0.036	0.2	0.0	-1038
			SIZE+DI+HOI	0.4	0.9	0.067	0.4	0.9	-1005
		growth	SIZE+DI	1.4	0.9	0.247	1.4	0.9	-27901
			SIZE+DI+HOI	1.0	0.9	0.260	1.0	0.9	-27703
	PA	survival	SIZE+DI	2.0	0.4	0.262	2.0	0.4	-115
			SIZE+DI+HOI	0.7	0.9	0.302	0.7	0.9	-109
		growth	SIZE+DI	0.9	1.1	0.316	0.9	1.1	-686
			SIZE+DI+HOI	1.0	1.0	0.336	1.0	1.0	-678

30

Table S2 Evaluations of model performance based on the parsimony tests and repeated k-fold cross validations (10 folds and 10 repeats) in case of optimum *u* and *v* and Radius = 10 m. Optimum *u* and *v* were selected for models with the highest R-squared and likelihood (Table S1). For the parsimony tests, AIC (Akaike's Information Criteria) and BIC (Bayesian Information Criteria) that were two or more points less than the next best model were considered as a meaningful improvement in in-sample performance. Models with lower RMSE (root mean square error) and MAE (mean absolute error) computed from cross validations had better out-of-sample performance. The numbers in bold indicated that HOIs-inclusive models had best performance based on AIC, BIC, RMSE or MAE.

Radius	Species	Response	Model	u	v	Para	Samples	R^2	Loglik	AIC	BIC	RMSE	MAE
10 m	Beech	Survival	SIZE	-	-	4	47560	0.007	-2250	4508	4543	0.090	0.016
			SIZE+DI	1.2	0.3	6	47560	0.009	-2246	4504	4557	0.090	0.016
			SIZE+DI+HOI	0.6	0.3	10	47560	**0.041**	**-2173**	**4367**	**4454**	**0.090**	**0.016**
		Growth	SIZE	-	-	2	35307	0.179	-43458	86923	86948	0.829	0.664
			SIZE+DI	1.1	0.6	4	35307	0.230	-42331	84671	84714	0.803	0.641
			SIZE+DI+HOI	0.9	0.7	8	35307	**0.238**	**-42148**	**84314**	**84390**	**0.799**	**0.638**
	Spruce	Survival	SIZE	-	-	4	1148	0.079	-258	523	544	0.241	0.118
			SIZE+DI	1.1	0.3	6	1148	0.189	-227	466	496	0.231	0.109
			SIZE+DI+HOI	0.8	0	10	1148	**0.222**	**-218**	**455**	506	**0.228**	**0.106**
		Growth	SIZE	-	-	2	753	0.193	-1043	2093	2107	0.966	0.778
			SIZE+DI	0.8	0.8	4	753	0.342	-967	1943	1966	0.874	0.693
			SIZE+DI+HOI	0.8	0.7	8	753	**0.355**	**-959**	**1936**	1978	**0.870**	**0.691**

Table S3 Evaluations of model performance based on the parsimony tests and repeated k-fold cross validations (10 folds and 10 repeats) in case of optimum *u* and *v* and Radius = 30 m. Optimum *u* and *v* were selected for models with the highest R-squared and likelihood (Table S1). For the parsimony tests, AIC (Akaike's Information Criteria) and BIC (Bayesian Information Criteria) that were two or more points less than the next best model were considered as a meaningful improvement in in-sample performance. Models with lower RMSE (root mean square error) and MAE (mean absolute error) computed from cross validations had better out-of-sample performance. The numbers in bold indicated that HOIs-inclusive models had best performance based on AIC, BIC, RMSE or MAE.

Radius	Species	Response	Model	u	v	Para	Samples	R^2	Loglik	AIC	BIC	RMSE	MAE
30 m	Beech	Survival	SIZE	-	-	4	31246	0.013	-1062	2133	2166	0.074	0.011
			SIZE+DI	0.2	0	6	31246	0.036	-1038	2089	2139	0.074	0.011
			SIZE+DI+HOI	0.4	0.9	10	31246	**0.067**	**-1005**	**2029**	**2113**	**0.074**	**0.011**
		Growth	SIZE	-	-	2	23245	0.182	-28875	57756	57780	0.838	0.673
			SIZE+DI	1.4	0.9	4	23245	0.247	-27901	55813	55853	0.804	0.641
			SIZE+DI+HOI	1	0.9	8	23245	**0.260**	**-27703**	**55425**	**55497**	**0.797**	**0.635**
	Spruce	Survival	SIZE	-	-	4	806	0.087	-143	293	312	0.204	0.088
			SIZE+DI	2	0.4	6	806	0.262	-115	242	270	0.195	0.079
			SIZE+DI+HOI	0.7	0.9	10	806	**0.302**	**-109**	**238**	285	**0.194**	**0.076**
		Growth	SIZE	-	-	2	535	0.181	-734	1474	1487	0.954	0.763
			SIZE+DI	0.9	1.1	4	535	0.316	-686	1382	1403	0.875	0.686
			SIZE+DI+HOI	1	1	8	535	**0.336**	**-678**	**1374**	1413	**0.869**	**0.684**

Table S4 Evaluations of model performance based on the parsimony tests and repeated k-fold cross validations (10 folds and 10 repeats) in case of optimum *u* and *v* and Radius = 20 m when categorizing neighbours as large trees (*DBH* > 10 cm) and small trees (*DBH* ≤ 10 cm)**.** Optimum *u* and *v* were selected for models with the highest R-squared and likelihood (Table S1). For the parsimony tests, AIC (Akaike's Information Criteria) and BIC (Bayesian Information Criteria) that were two or more points less than the next best model were considered as a meaningful improvement in in-sample performance. Models with lower RMSE (root mean square error) and MAE (mean absolute error) computed from cross validations had better out-of-sample performance. The numbers in bold indicated that HOIs-inclusive models had best performance based on AIC, BIC, RMSE or MAE.

Radius	Species	Response	Model	*u*	*v*	Para	Samples	R^2	Loglik	AIC	BIC	RMSE	MAE
20 m	Beech	Survival	SIZE	-	-	4	38798	0.011	-1433	2874	2908	0.078	0.012
			SIZE+DI	0.8	0.0	6	38798	0.029	-1408	2827	2878	0.078	0.012
			SIZE+DI+HOI	0.5	0.7	10	38798	**0.067**	**-1352**	**2724**	**2809**	**0.078**	**0.012**
		Growth	SIZE	-	-	2	28845	0.180	-35775	71557	71582	0.836	0.671
			SIZE+DI	1.5	0.8	4	28845	0.244	-34611	69231	69273	0.803	0.640
			SIZE+DI+HOI	1.5	0.8	8	28845	**0.267**	**-34156**	**68330**	**68405**	**0.791**	**0.630**
	Spruce	Survival	SIZE	-	-	4	1058	0.099	-239	486	506	0.244	0.121
			SIZE+DI	1.2	0.4	6	1058	0.212	-209	431	461	0.232	0.110
			SIZE+DI+HOI	1.3	0.4	10	1058	**0.305**	**-185**	**389**	**439**	**0.230**	**0.105**
		Growth	SIZE	-	-	2	692	0.181	-967	1941	1954	0.979	0.787
			SIZE+DI	1.0	1.1	4	692	0.308	-909	1828	1851	0.902	0.711
			SIZE+DI+HOI	1.4	0.9	8	692	**0.336**	**-894**	**1807**	**1848**	**0.889**	**0.710**

Figure S1 The spatial distribution of *Fagus sylvatica* (Beech, red) and *Picea abies* (Spruce, blue) within the 25-ha Zofin plot.

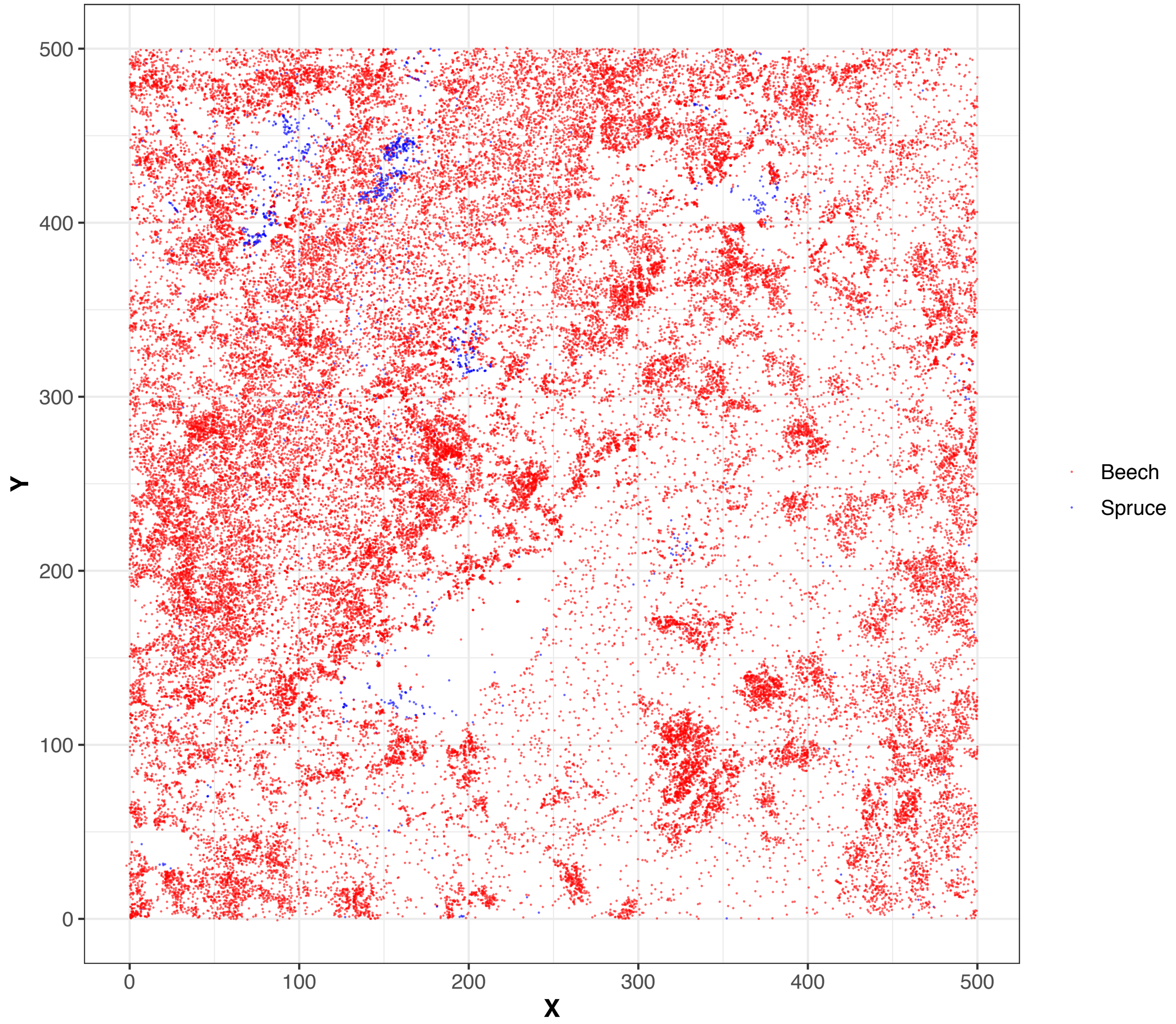

Figure S2 Neighbourhood direct (DI) and higher-order effects (HOI) decay as a function of distance. The shapes of the curves are determined by *v* (ranging from 0 to 1) and are truncated at the maximum radius (R = 30 m).

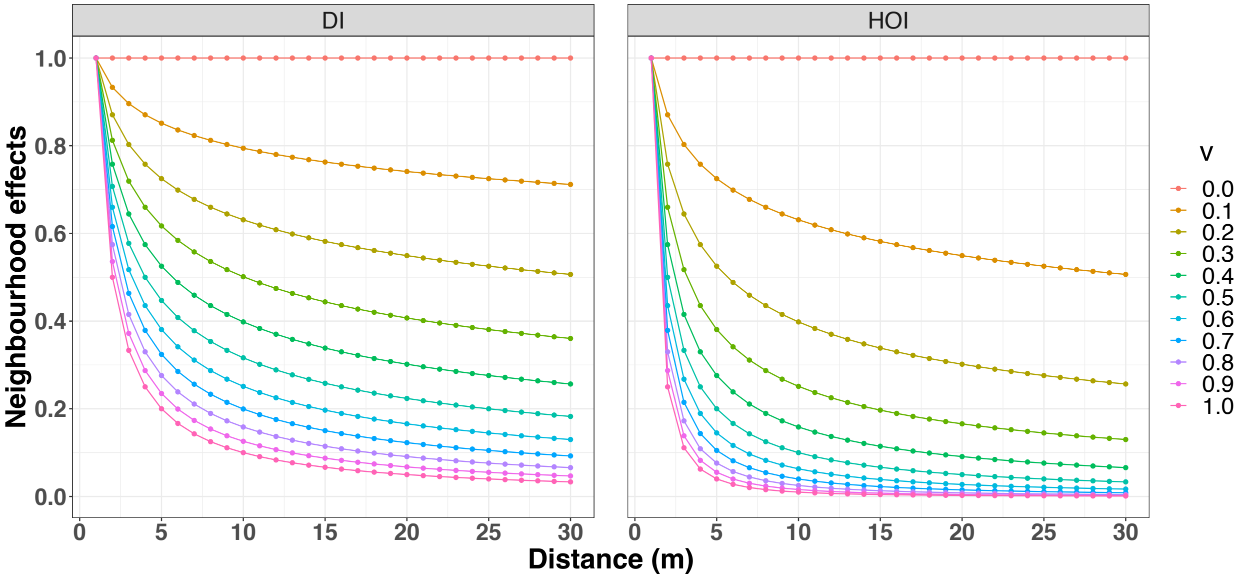

Figure S3 The *DBH* distribution of *Fagus sylvatica* (Beech) and *Picea abies* (Spruce). Vertical red line indicates the threshold (*DBH* = 10 cm) used to categorise neighbours into large trees and small trees. Small trees constitute the majority (90% for Beech and 66% for Spruce) of both species.

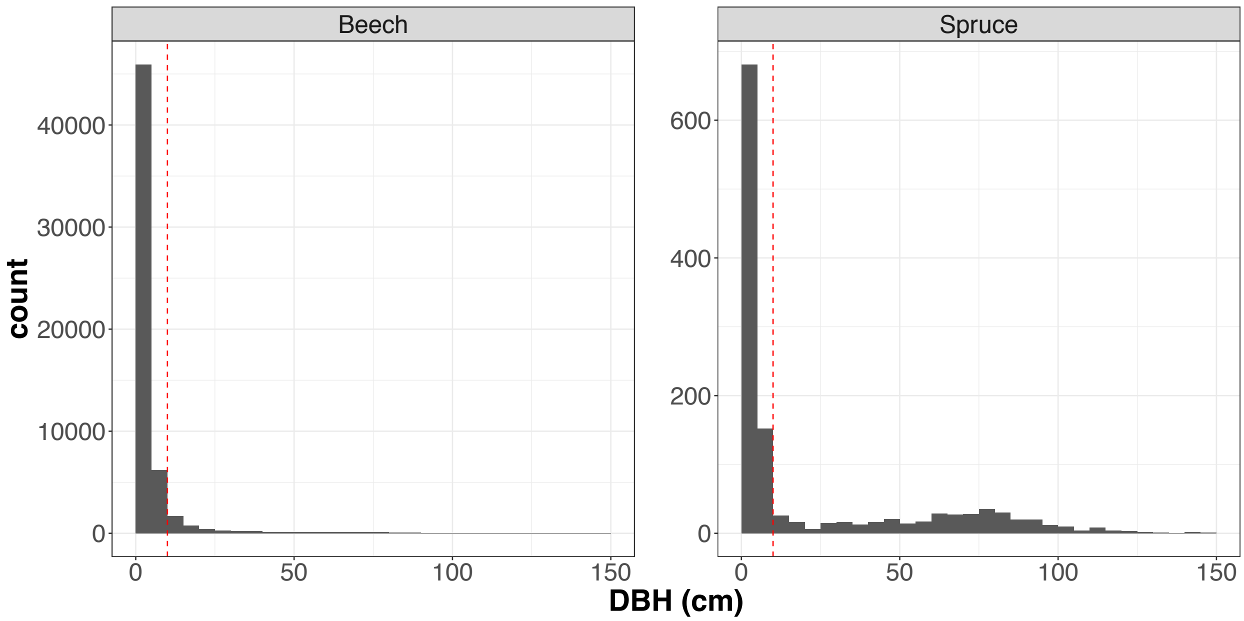

Figure S4 The spatial distribution of large trees (L, *DBH* > 10 cm, red) and small trees (S, *DBH* ≤ 10 cm, blue) within the 25-ha Zofin plot.

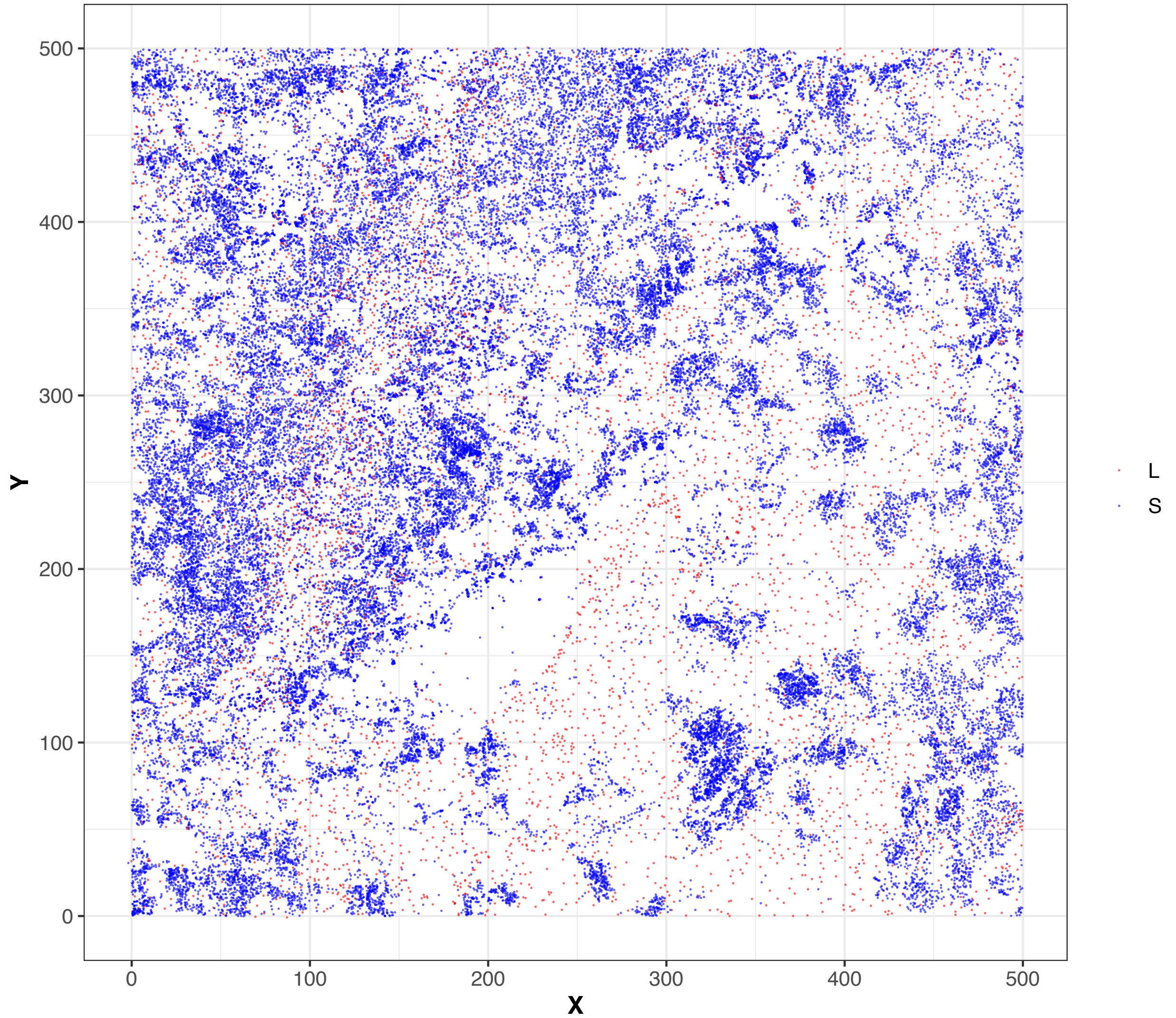

Figure S5

Cumulative (purple), direct (red) and higher-order (blue) effects of on the survival (a and c) and growth (b and d) of each focal tree of *Fagus sylvatica* (Beech, a and b) and *Picea abies* (Spruce, c and d) when categorizing neighbours as large trees ($DBH > 10$ cm) and small trees ($DBH \leq 10$ cm). Total indicates cumulative effects of all neighbours including both direct and higher-order effects. DI includes the direct effects of all neighbours including direct effects of large trees (DI_{iL}) and direct effects of small trees (DI_{iS}). HOI includes higher-order effects of large trees on focal tree in the presence of other large trees ($HOI_{iL,L}$), higher-order effects of large trees on focal tree in the presence of small trees ($HOI_{iL,S}$), higher-order effects of small trees on focal tree in the presence of large trees ($HOI_{iS,L}$), and higher-order effects of small trees on focal tree in the presence other small trees ($HOI_{iS,S}$). One boxplot represents the distribution of neighbourhood effects for all focal trees of a species. Boxplots above (or below zero) indicate the effects are facilitative (or competitive) for all trees of a species, while boxplots crossing the zero line indicate the effects are facilitative for some trees but competitive for others of a species.

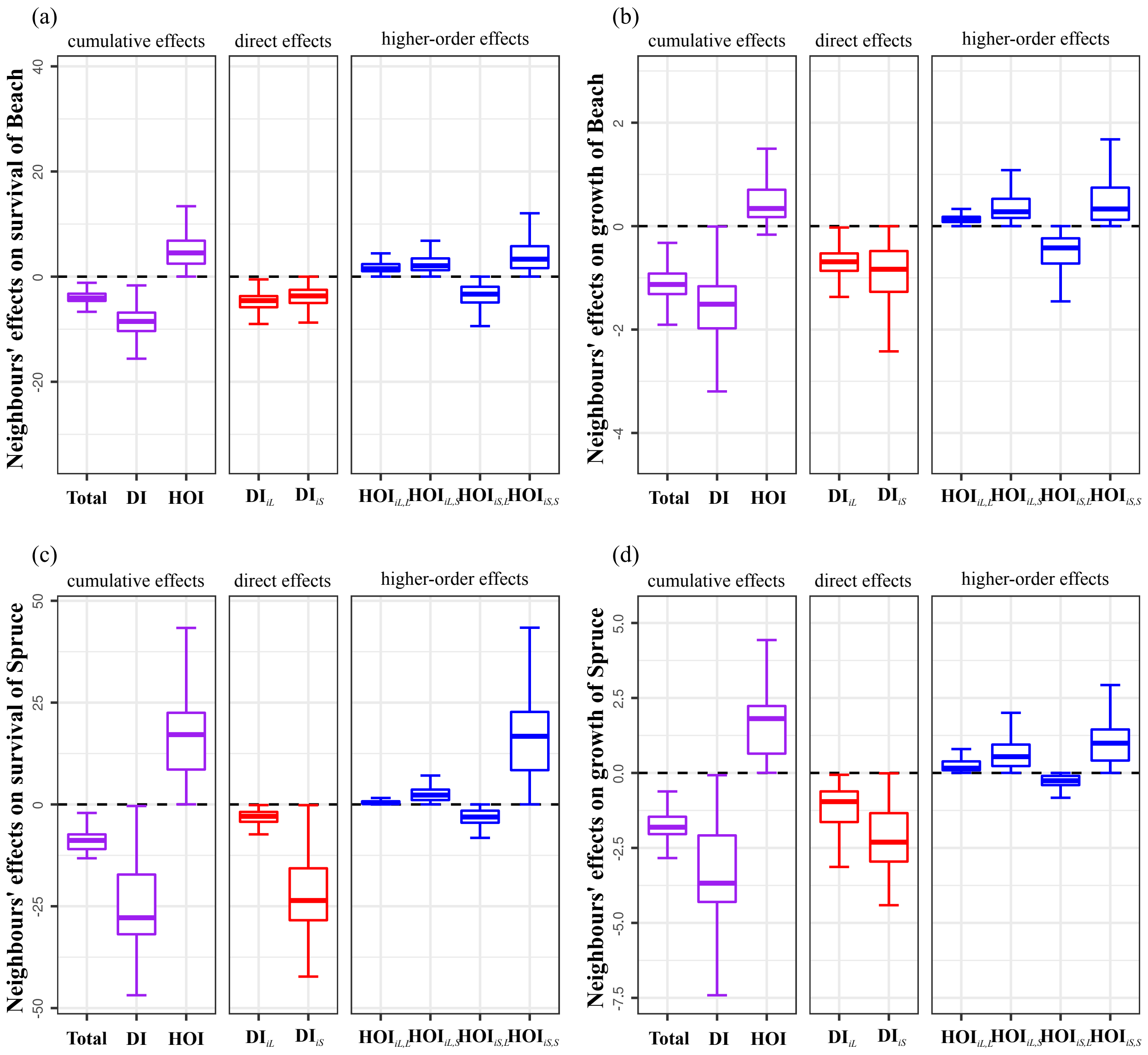
(a)
cumulative effects
direct effects
higher-order effects
Neighbours' effects on survival of Beach
Total
DI
HOI
(b)
Neighbours' effects on growth of Beach
(c)
Neighbours' effects on survival of Spruce
(d)
Neighbours' effects on growth of Spruce
